# Points in Regard to Army Legislation

**Published:** 1898-06

**Authors:** 


### POINTS IN REGARD TO ARMY LEGISLATION.

Pennsylvania has pledged herself to raise two hundred dollars for expenses of representatives at Washington in the interest of those of our colleagues in the army who are without rank.

Mr. Daniel O'Driscol, of Washington, D. C., represents in a measure the army veterinarians. It might be a good point for the President of the U. S. V. M. A. to delegate the senior editor of the JOURNAL, Dr. Huidekoper, for special services in this direction.

Hon. James Wilson, Secretary of Agriculture, is much interested in the recognition of the veterinarian in the army, and has expressed himself through public journals strongly in favor of the movement destined to give the army veterinarian rank.

Army veterinarians of ten years' service should certainly have much accorded them by reason of this period, when the same has been satisfactory.

Dr. J. P. Turner, of the Sixth United States Cavalry and member of the Army Legislative Committee, has resigned from the service, taken the civil-service examination, and entered the meat-inspection service, and has been assigned to duty at St. Louis, Mo.

Information reaches us that many farriers and other insufficiently educated men are being assigned to perform veterinary services in the rapidly increasing army. Surely this is heaping insult upon insult to a profession that has rendered such important work for our government. Have the authorities at Washington forgotten that the veterinary profession of this country enabled our government to stamp out contagious pleuro-pneumonia, el dourine, and are rapidly developing plans for the radical control of bovine tuberculosis and hog-cholera? Ingratitude is the highest offence our governing authorities could return for such labors and results.

